# New components of the *Dictyostelium *PKA pathway revealed by Bayesian analysis of expression data

**DOI:** 10.1186/1471-2105-11-163

**Published:** 2010-03-31

**Authors:** Anup Parikh, Eryong Huang, Christopher Dinh, Blaz Zupan, Adam Kuspa, Devika Subramanian, Gad Shaulsky

**Affiliations:** 1 Graduate program in Structural Computational Biology and Molecular Biophysics, Baylor College of Medicine, One Baylor Plaza, Houston, TX 77030, USA; 2 Department of Molecular and Human Genetics, Baylor College of Medicine, One Baylor Plaza, Houston, TX 77030, USA; 3 Department of Biochemistry and Molecular Biology, Baylor College of Medicine, One Baylor Plaza, Houston, TX 77030, USA; 4 Faculty of Computer and Information Science, University of Ljubljana, Trzaska cesta 25, SI-1001 Ljubljana, Slovenia; 5 Department of Computer Science, Rice University, 6100 Main St, MS 132, Houston, TX 77005, USA

## Abstract

**Background:**

Identifying candidate genes in genetic networks is important for understanding regulation and biological function. Large gene expression datasets contain relevant information about genetic networks, but mining the data is not a trivial task. Algorithms that infer Bayesian networks from expression data are powerful tools for learning complex genetic networks, since they can incorporate prior knowledge and uncover higher-order dependencies among genes. However, these algorithms are computationally demanding, so novel techniques that allow targeted exploration for discovering new members of known pathways are essential.

**Results:**

Here we describe a Bayesian network approach that addresses a specific network within a large dataset to discover new components. Our algorithm draws individual genes from a large gene-expression repository, and ranks them as potential members of a known pathway. We apply this method to discover new components of the cAMP-dependent protein kinase (PKA) pathway, a central regulator of *Dictyostelium discoideum *development. The PKA network is well studied in *D. discoideum *but the transcriptional networks that regulate PKA activity and the transcriptional outcomes of PKA function are largely unknown. Most of the genes highly ranked by our method encode either known components of the PKA pathway or are good candidates. We tested 5 uncharacterized highly ranked genes by creating mutant strains and identified a candidate cAMP-response element-binding protein, yet undiscovered in *D. discoideum*, and a histidine kinase, a candidate upstream regulator of PKA activity.

**Conclusions:**

The single-gene expansion method is useful in identifying new components of known pathways. The method takes advantage of the Bayesian framework to incorporate prior biological knowledge and discovers higher-order dependencies among genes while greatly reducing the computational resources required to process high-throughput datasets.

## Background

Cellular function depends on the coordination of thousands of genes whose expression and activities are regulated by complex networks. Understanding these networks is essential for elucidating cell function, and is a central question in systems biology. PKA (cAMP-dependent protein kinase) is an important regulator of cellular function in many eukaryotes. The role of PKA in development has been studied extensively in the amoeba *Dictyostelium discoideum *using biochemistry, genetics and cell biology, but the underlying transcriptional regulatory network remains largely unknown. For example, one of the most important missing components is CREB (cAMP-response element-binding protein), the bZIP transcription factor that couples cAMP signaling with gene expression in most eukaryotes [1]. We have used gene-expression data from thousands of experiments to improve our understanding of PKA regulation and to uncover new components in the network.

**Figure 1:**
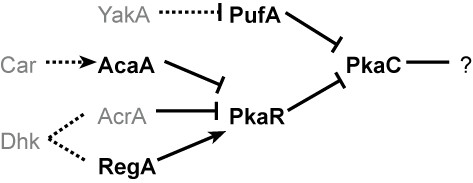
**The PKA-regulatory pathway**. Biochemical, genetic and physiological data were used to describe a pathway that regulates PKA during *Dictyostelium *development. Gene expression data were not considered in the construction of this network. PufA is an RNA-binding protein that sequesters *pkaC *mRNA and prevents its translation. YakA is a protein kinase that indirectly inhibits PufA activity. PkaC catalytic activity is inhibited by the regulatory subunit PkaR. An extracellular cAMP signal is integrated through various cAMP receptors (Car), which result in the activation of the aggregative adenylyl cyclase AcaA. AcaA activation leads to production of cAMP, which binds PkaR and ends the inhibition of PkaC. Two proteins that contain response regulator domains control PkaR. The adenylyl cyclase AcrA functions after the aggregation stage of development and produces cAMP, and the cAMP-phosphodiesterase RegA degrades intracellular cAMP. These two response regulator proteins are regulated indirectly by histidine kinases (Dhk). The components that function downstream of PkaC are unknown (?). Nodes in the graph represent genes and edges represent regulatory relationships: positive (arrows) and negative (barred lines). Dashed lines indicate indirect interactions. The proteins in black are encoded by genes used in our analysis.

*D. discoideum *cells are free-living soil amoebae that prey on bacteria and propagate as single-celled organisms when food is abundant. Upon starvation, the cells aggregate, differentiate into 2 types and form fruiting bodies that consist of balls of spores carried atop cellular stalks [2]. The control of cAMP synthesis and the regulation of PKA are essential for the transition from growth to development and for all subsequent developmental stages (Figure 1). Mutations in genes of the PKA pathway cause severe developmental defects. Elimination of positive regulators results in lack of aggregation and elimination of negative regulators causes precocious development [3]. Genome-scale analysis of the *D. discoideum *PKA regulatory network should help to identify pathway components and reveal emergent properties that may predict novel network behavior.

Recently, many techniques to analyze gene-expression patterns have been suggested. Methods using clustering or correlation [4-6] have fallen short of uncovering the complex dependences governing regulatory networks. Many probabilistic graphical approaches, using probabilistic Boolean networks, information theory, and Bayesian networks, have been used to model the connectivity of regulatory networks. In a probabilistic Boolean network, a gene state is predicted from the state of several other genes by a set of probabilistic functions [7]. Information theory approaches, such as ARACNE, compare expression profiles between all genes using mutual information as a generalized measure of correlation [8]. Bayesian networks are useful because they can model higher than pairwise orders of dependences between genes and can incorporate existing knowledge [9-11]. They have been used to learn direct, causal dependencies among genes from expression data, distinguishing them from simple correlations [12]. Unfortunately, a major limitation of Bayesian network algorithms is their inability to model cyclic networks. Furthermore algorithms that infer network structure *ab initio *from experimental data scale super-exponentially with the number of variables (genes), so restrictive assumptions must be made for computational feasibility. State-of-the-art algorithms that provide exact results can only handle networks with 30-50 genes [13], while heuristic approaches often require strong assumptions [14], and rely on the availability of very large, high quality datasets that represent a wide range of states for each gene in the network. The paucity of such datasets in biology makes network structure inference challenging.

## Methods

### Microarray data

We created a microarray data management system for 2,495 experiments that consists of gene-expression data from various strains and mutants grown under many different conditions. The microarrays represent 4,053 *D. discoideum *genes [15]. Our data management and analysis pipeline incorporates the LIMMA package [16] in R/BioConductor for quality control and normalization. Since these data are derived from different experiments under different conditions, we implemented a normalization algorithm, which accounts for variation within and between experiments [17], and a filtering schema to reject low quality data (data with low correlation between on-chip replications). Individual chips with low correlation between duplicate spots and duplicate spots with low correlation across all chips were removed. Chips passing the quality filter were normalized using the "printtip loess" normalization function in LIMMA, followed by median scaling. Print tip normalization accounts for signal intensity biases introduced during the array printing process, while loess normalization removes the biases introduced by different labeling dyes [18]. Next the data are median-scaled to account for differences in hybridization kinetics across experiments. Finally the expression data are merged into a unified dataset to allow meta-analysis. To deal with the inherent noise in gene expression data, the expression values were discretized into three categories: under-expressed, normal and over-expressed, as compared to the average across all experiments [19]. We tested multiple discretization strategies and found they made little difference to the final Bayesian analysis. The entire dataset is available at http://www.ailab.si/dictyexpress/data.htm.

### Bayesian networks

We used Bayesian networks [20] to model the core PKA pathway (Figure 1). A Bayesian network encodes a pathway as a joint probability distribution over variables denoting the expression levels of all the genes in that pathway. The network is a factored, graphical representation of the full joint distribution over the expression levels. The graph structure of the network encodes conditional independence relationships between the genes. More formally, a Bayesian network over a set V = {V_1_,..., V_n_} of n genes is a pair (G, θ), where G is a directed acyclic graph whose vertices represent the variables V_1_,..., V_n_, and whose edges represent direct dependencies between the variables and θ represents the set of conditional probability distributions of the form P(V_i_|Parents(V_i_, G)), for all i = 1,..., n. The qualitative part of the model is the topology of the G, while the quantitative part is the set θ of local conditional probability distributions. The full joint probability distribution P(V_1_,..., V_n_) can be reconstructed as the product of the individual conditional probability distributions in θ.

### Learning Bayesian networks and the Single-gene expansion strategy

The problem of learning a Bayesian network from data is posed as an optimization problem: given a data set D = {v|v € R^n ^} of m joint measurements of n genes, find a network B* = (G*, θ*) which maximizes the posterior probability of the network given the data.

The first term P(D|G, Θ) is the likelihood of the expression data D given the network (G, θ), the second term is the probability of the parameters Θ given the graph structure, and the third term is the prior probability of the graph G. To compute the posterior probability of a graph G with respect to the data set D, we assume a uniform graph prior P(G), and a Dirichlet prior [11] for P(θ|G). The best network with respect to the data is one that maximizes the posterior probability P(G|D). The logarithm of P(G|D) is called the Bayesian score of the network. Finding the network with the highest Bayesian score is known to be NP-complete [11]. There are two heuristic approaches used to finding approximate solutions to the combinatorial optimization problem - direct search for a graph G guided by the Bayesian scoring function, and using Markov chain Monte Carlo sampling of graphs from the posterior distribution P(G|D). In this paper, we propose a modification of the direct search procedure described in [10]. Instead of starting *ab initio*, we began with a known network, including the member genes and their connectivities and extend, one gene at a time, from a genome wide expression data set. We call this the *single-gene expansion strategy*. Starting with a core pathway of 5 genes in the PKA pathway (Figure 1), we expanded the network by adding a single gene at a time in all possible ways that preserve the acyclicity of the expanded network. In all, up to 2^10 ^networks were considered for each gene, corresponding to all possible ways the gene can be added to the core network. Then we calculate the Bayesian score of each expanded 6-node network. The highest scoring 6-node network represents the likelihood that the inserted gene is involved in the PKA pathway. The score associated with the gene, called the Bayesian addition score, is the difference between the Bayesian score of the best 6-node network with the gene, and the Bayesian score of the core pathway (i.e., without the gene). The rank of each inserted gene is determined by its Bayesian addition score, which was computed as follows:

where *D *is the set of high quality data for the genes in the network, and G is the core PKA network. We chose not to perform cross-validation, since this analysis would require random sub-sampling of the expression dataset resulting in the exclusion of different perturbation experiments. Modeling the data subsets would produce highly variable sub-optimal Bayesian addition scores that do not reflect the most likely model given the entire dataset.

The single-gene expansion strategy and the computation of the Bayesian addition scores was implemented using the Bayes Net Toolbox for Matlab [20]. The Matlab script we used is provided in Additional file 1. Bayesian addition scores were calculated for 4,053 genes in our expression data set.

### Co-expression analysis

As a simpler alternative to the proposed technique we have considered co-expression analysis, which does not take into account a prior knowledge of network structure and instead only considers pairwise interactions between genes. We used correlation between expression levels of a given gene and the genes in the core pathway as a measure of its relevance to the network. In particular, we defined a co-expression score of a gene as the minimum pairwise distance between the gene and every member of the core network.

The co-expression score was used to rank the 4,053 genes in our expression data set.

There are many distance measures that can be used, including Pearson correlation and Euclidean distance. While Euclidean distances are very sensitive to the magnitudes of expression levels, Pearson correlations are more robust since they measure the strength and direction of a (linear) relationship between expression levels. Therefore, we chose to use Pearson correlation over Euclidean distance in our analysis. The co-expression score of a new gene is the highest Pearson correlation between the gene and each of the other genes in the core network.

### Statistical analyses

Hypergeometric distribution (Phyper function in the statistical software package R) was used to determine enrichment of developmental genes among the top-ranked genes with published phenotypes. All published mutants and their phenotypes are available at http://www.dictybase.org. We used a 1-sided, unequal variance Student's *t*-test (t.test function in the statistical software package R) to examine whether the expression values of some of the genes were characterized by higher variability than the other genes.

### GST-fusion protein and EMSA in vitro

The bZIP region of *bzpF*, coding for the DNA-binding domain and the leucine zipper domain, was cloned into the pGEX4T1 vector (Amersham Biosciences) upstream of GST to generate a GST-fusion protein. The construct was verified by restriction analysis and sequencing and transformed into *E. coli *strain BL21star (Invitrogen). Gene expression was induced with IPTG and the protein was purified on glutathione-sepharose beads (Amersham Biosciences) according to the manufacturer's recommended protocol. The protein was used in an electrophoretic mobility shift assay (EMSA) [21] with 2 double-stranded oligonucleotides. The CRE-containing oligonucleotide was 5' AGC TAA TAT GAG AAA AT**T GAC GTC A**TT AAC TTT T 3' (the CRE sequence is shown in bold letters), and the CRE-negative oligonucleotide was 5' AGC TAA TAT GAG AAA AT**T CAC AAAA**TT AAC TTT T 3', (mutations of the CRE sequence are underlined). The oligonucleotides were annealed with complementary oligonucleotides (5' AAA AGT TAA TGA CGT CAA TTT T 3' and 5' AAA AGT TAA TTT TGT GAA TTT T 3', respectively) and labeled radioactively by filling in with Klenow fragment of DNA polymerase (Invitrogen) in the presence of α-^32^P-dATP. The labeled oligonucleotides were mixed with the purified protein at room temperature, incubated for 30 minutes and resolved by electrophoresis through a native 5% polyacrylamide gel in 0.5× TBE buffer at 200 volts for 2 hours. The gels were dried under reduced pressure and autoradiography was performed to visualize the binding products.

## Results

We extended the Bayesian network framework to facilitate an exploratory analysis of specific pathways to identify new potential members. We started by testing whether transcriptional profiles could provide information for reconstruction of a regulatory network. We analyzed 2,495 expression-array experiments, consisting of data on 4,053 genes, including 5 genes from the established PKA pathway (Figure 1). The pathway was constructed without consideration of gene expression, so there was no reason to assume that it could be reconstructed from expression data. Nevertheless, we chose this network for several reasons, not the least of which is its biological significance. Firstly, we assumed that it would provide a more rigorous test of the approach than the analysis of a known transcriptional network. Moreover, the network includes cases in which two genes coordinate the expression of a third gene, but the two are not necessarily coordinately regulated. We postulated that incorporating prior knowledge from other sources would allow better identification of potential pathway members that depend on two or more core members.

**Figure 2:**
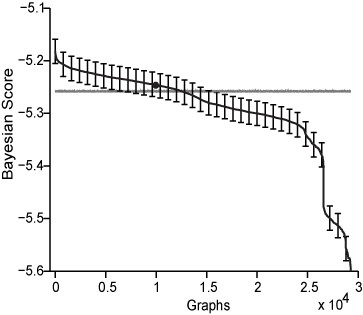
**The distribution of Bayesian likelihood scores**. We calculated the Bayesian scores (y-axis) of all possible 29,281 5-node networks (Graphs, x-axis) from the intact data (black line) and from randomized data (grey line), sorted them and plotted them from best to worst. The known biochemical network (black dot) ranks 10,313. Standard deviations were calculated for each point using bootstrap analysis. For clarity, error bars were plotted at intervals of 800.

The transcriptional data we used were from cells with different genotypes that were subjected to various growth and developmental conditions [15,22-28]. All the data are available in various public repositories, but we have also collected and deposited them in our repository for added convenience http://www.ailab.si/dictyexpress/data.htm. We used the Bayesian scoring function to evaluate all possible networks connecting the 5 genes. As a control, the same networks were scored with randomly shuffled expression data (Figure 2). We found that the network scores obtained from the intact data were variable, whereas the network scores obtained from the shuffled data were nearly indistinguishable, indicating that Bayesian modeling is capable of extracting significant biological information from this domain. The rank of the known 5-gene network was 10,313, which is not significantly different from the rank obtained from the shuffled data (Figure 2). This finding was expected because the pathway was constructed without consideration of transcriptional data and because the 5 genes are not coordinately regulated. This finding is not related to the applicability of our algorithm, since our goal is to discover potential new members of a known pathway, rather than to rediscover the topology of a known pathway. Additionally, since mRNA levels do not necessarily correlate with protein levels and protein function, we use transcriptional data only as a surrogate measure to discover new members of the PKA pathway. To find new pathway members, we implemented a single-gene expansion approach. We expanded the PKA pathway, which includes 5 genes and their connectivities (Figure 1), by adding a single new gene at a time to make all possible 6-node networks. We scored the new networks by calculating the Bayesian addition scores, e.g. the difference between the Bayesian score of the augmented network and that of the core network.

**Figure 3:**
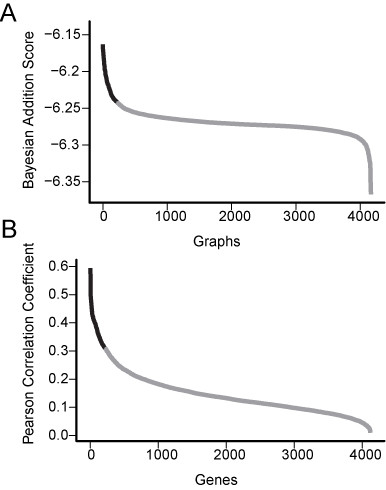
**Single-gene expansion reveals candidate pathway components**. We compared the distribution of rankings from the single-gene expansion method and co-expression analysis. A. Genes were added to the network one at a time in all possible putative positions and the Bayesian addition scores (y-axis) were calculated for each new network (x-axis). The top scores for each gene were plotted from best to worst (grey line). The top 5% of the distribution (black line) consist of 209 genes. B. Similarity between genes and network members was computed by calculating all pairwise Pearson's correlations and the maximal correlation score (y-axis) was used to rank each new gene (x-axis). Using the cutoff determined by the single-gene expansion method, we focused our comparison on the top 5% of the distribution (black line).

The scores determine the rank of the inserted gene among all genes in the dataset - a higher rank indicates a higher conditional dependence between the new gene and the PKA network, as a whole. Figure 3A shows the distribution of the Bayesian addition scores for all the genes. The near-horizontal center of the sigmoid curve shows that most genes are not significantly different from each other in their effect on the network. A few genes received high scores, suggesting significant relationships with the network (the left part of the plot) and a few received low scores (the right part of the plot). The latter is due to low-quality data, since the expression values were characterized by high variability compared to the other genes (Student's *t*-test p-value 4 × 10^-14^). These findings suggest that only a few new genes may be involved in the PKA pathway. We therefore focused our analysis on the 209 genes that ranked as the top 5% (black line, Figure 3A). This group contains mostly novel genes without definable domains and several genes of known or presumed function (Additional file 2, Table S1).

Mutations in known PKA-pathway genes cause developmental defects [27]. We postulated that mutations in new pathway genes would also cause such phenotypes. The *Dictyostelium *genome is sparsely annotated - only 433 genes have known or presumed function. Despite this sparse annotation, we find that 13 of the 209 top ranked genes have been characterized previously, representing a statistically significant enrichment of genes with known or presumed function (Hypergeometric Test, p-value 0.005). Of the 13 previously characterized genes, previous work has shown that 12 are essential for proper development (Table 1). One possible explanation for this finding could have been that published work is skewed in favor of mutants with developmental abnormalities. However, we calculated that only 67% of all the characterized mutants in *D. discoideum *have developmental abnormalities. Thus, the enrichment we observed of genes essential for development is significantly higher than that publication skewing (Hypergeometric Test, p-value 0.002). The published data suggest that most of the 12 genes are involved in the PKA network (Table 1), validating the single-gene expansion approach.

**Table 1:** Retrospective analysis of high-ranking genes

Bayesian Rank	Co-exp Rank	Gene name	Mutant phenotype^a^	Reference
4	16	*dhkK*	Aberrant slug migration	[40]
33	31	*gbfA*	Development arrests at mound stage	[41]
42	69	*wimA*	Aberrant fruiting body morphology	[42]
77	44	*dhkC*	Precocious development	[43]
79	8	*comC*	Aberrant aggregation	[44]
92	174	*rzpA*	Aberrant aggregation	[45]
112	NA	*egeB*	Development arrests at mound stage	[37]
115	NA	*Sel1*-like	Wild Type	[42]
118	138	DG1037	No aggregation	[42]
122	208	*mybE*	Increased slug size	[46]
123	157	*CRTF*	No aggregation	[47]
140	178	*cudA*	Development arrests at slug stage	[48]
189	214	*tsg101*	Small fruiting body	[49]
204	NA	*cbpC*	Delayed culmination	[50]

NA - Not ranked in the top 5% of the distribution; a - Mutant phenotypes were obtained from http://dictybase.org/. Twelve of the 13 high-ranking genes with published mutant phenotypes exhibit developmentally abnormal phenotypes when mutated.

To test whether the single-gene expansion method has an advantage over a simpler approach of identifying co-expressed genes, we subjected the data to a similar analysis using co-expression networks instead of Bayesian networks. Unlike co-expression analysis, our addition procedure goes beyond considering pairwise interactions between the new gene and the existing network, and includes all potential n-ary interactions to judge the relevance of the gene to the PKA pathway. Therefore we expected the single-gene expansion method performance to be comparable to co-expression networks when genes have simple pairwise dependences, but to have an advantage in discovering genes with higher-order dependences in the core network. We measured the pairwise similarity of each new gene to each of the 5 PKA pathway genes using Pearson's correlation. Figure 3B show the distribution of co-expression scores for all the genes. The shape of the curve we observed is also sigmoid but it lacks distinct groups of high and low likelihood scores. This observation suggests that the co-expression method was less efficient in distinguishing potential network members from unrelated genes at the high end, and performed very poorly on genes with low quality data at the low end. Therefore, we used the cutoff identified using the single-gene expansion method and focused our comparison on the top 5% ranked genes by both analyses. We found that 133 of the top-ranking genes were discovered by both methods (Additional file 2, Table S1 and Additional file 3, Table S2), suggesting that many of the 209 top-ranked genes have relatively simple pairwise dependences with one of the genes in the 5-gene network. The remaining 76 genes not revealed by the pairwise analysis, therefore, are likely dependent on 2 or more genes in the core network. To test that possibility we computed the number of dependences on core network genes for each of the 76 genes discovered only by the single-gene expansion method. We found that the number of dependences on core network genes was significantly higher for those 76 genes compared to the genes found by both methods (Students *t*-test, p-value 0.01) (Additional file 2, Table S1). While the single-gene expansion method identifies higher-order dependences, this analysis does not detect the strength of the dependences.

To further validate our approach, we tested additional *Dictyostelium *strains with mutations in genes from the top 5% whose developmental roles were unknown (Table 2). Our selection criteria included availability of knockout vectors from the Functional Genomics Project at Baylor College of Medicine [29], and the ability to successfully generate and grow the knockout strains. We successfully created 5 mutant strains, one with a disruption in a histidine kinase gene (*dhkL*), which is likely to have a role upstream of PKA, 3 in basic leucine-zipper transcription factors (bZIP) that are potential CREB homologs, predicted to function downstream of PKA, and one with no sequence homology.

**Table 2:** Experimental validation of predictions

Bayesian Rank	Co-exp Rank	Gene name	Mutant phenotype
34	92	*bzpR*	None observed^a^
35	33	*dhkL*	Precocious development^b^
98	NA	BC5V2_0_00231	None observed^c^
166	NA	*bzpG*	None observed^a^
188	129	*bzpF*	Aberrant fruiting bodies^a^

NA - Not ranked in the top 5% of the distribution, a - Huang and Shaulsky, unpublished; b - this study (Figure 5); c - data not shown.

**Figure 4:**
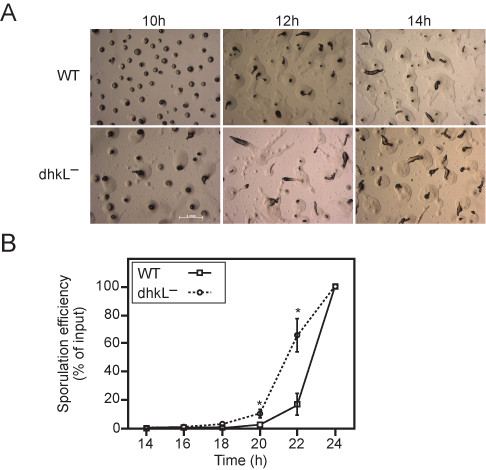
***dhkL*^- ^cells exhibit accelerated development**. Wild-type (WT) and *dhkL*^- ^cells were developed on buffered agar. A. Developmental morphology at 10, 12 and 14 hours as indicated. Bar - 1 mm. The *dhkL*^- ^mutants were indistinguishable from the wild type cells during the first 8 hours of development (data not shown). At 10 hours, the mutants formed fingers and tipped aggregates, while the wild-type cells only formed tight aggregates. At 14 hours, the mutants progressed to the slug stage, while the wild-type cells just entered the finger stage. After 20 hours, the mutants resembled the wild type cells again (data not shown). B. We counted the number of spores at 2-hour intervals during development of *dhkL*^- ^(circles and dashed lines) and wild type cells (squares and solid lines). The sporulation efficiency (% of cells that became spores) is plotted as a function of time (14-24 hrs), the average and standard error from 3 independent replications. Error bars are not shown when smaller than the symbol. Asterisks indicate a significant difference between the wild type and the mutant values (Student's t-test, p < 0.05).

We mutated the *dhkL *gene and found that the mutants exhibited accelerated mid-development progression (Figure 4A). The *dhkL*^- ^mutants showed a marked acceleration in development at 10 hours, but by 20 hours they resembled the wild type again. To quantify this phenotype we developed wild-type and *dhkL*^- ^mutant cells and counted the number of spores during development. The mutants started to form spores 2 hours before the wild type, and made 3-fold more spores at 18 hours of development. That difference decreased to 2-fold at 20 hours and disappeared by 22 hours (Figure 4B). Mutations in several PKA-pathway genes cause rapid development [3], so the observed phenotypes suggest that *dhkL *is indeed a member of the PKA pathway, probably functioning as a negative regulator. Histidine kinases function by phosphorylating response regulators. The *D. discoideum *genome encodes two known response regulators [30] - the cAMP phosphodieasterase *regA *and the adenylate cyclase *acrA *[31,32]. Thus the function of *dhkL *in the PKA pathway may be mediated by these response regulators, which modulate cAMP levels directly.

**Figure 5:**
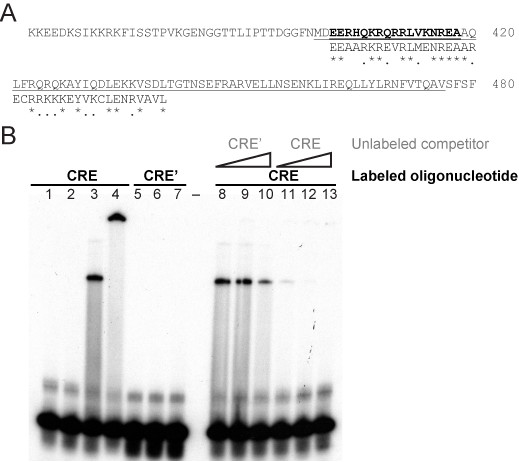
**BzpF is a candidate CREB homolog, which binds CRE DNA in vitro**. A. The 120-amino-acid region of BzpF, containing the conserved basic-leucine-zipper region is shown (aa 360-480). Amino acids in bold correspond to the CRE-binding motifs in other eukaryotes as shown in alignment with the human CREB (NP_604391, aa 280-318). The underlined region was expressed as a GST-fusion protein in *E. coli *and used for EMSA. B. The purified BzpF-GST fusion protein was used in EMSA with radioactively labeled oligonucleotides containing the canonical CRE motif (CRE, lanes 1-4) or with a mutated form of CRE (CRE', Lanes 5-7). An autoradiogram is shown. Lanes 1 and 5 - no protein added; lanes 2 and 6 - GST protein alone (without the BzpF fusion); lane 3 and 7 - BzpF-GST fusion protein; lane 4 - BzpF-GST fusion protein with anti-GST antibodies added for super shift. Competition EMSA was done under the same conditions as in Lane 3 but in the presence of increasing amounts of unlabeled CRE' (Lanes 8-10) or CRE (Lanes 11-13) oligonucleotides as indicated above the lanes. The triangles indicate increased ratios of labeled to unlabeled oligonucleotides--1:10, 1:50 and 1:500). Black text indicates the labeled oligonucleotides and grey text indicates the unlabeled competitor.

Previous efforts have failed to identify CREB homologues among the 19 bZIPs in the *Dictyostelium *genome [30], but our analysis implicated three bZIPs as potential PKA pathway components (Additional file 2, Table S1). Sequence analysis revealed a degenerate cAMP-response element (CRE) binding motif only in *bzpF*, implicating it as a potential CREB (Figure 5A). We examined the ability of the BzpF protein to bind the canonical CRE by expressing a GST-fusion protein in bacteria and testing it in an electrophoretic mobility shift assay. We found that the fusion protein bound a CRE-containing oligonucleotide (Figure 5B, lane 3) but not a mutated oligonucleotide (Figure 5B, lane 7), suggesting that BzpF can bind CRE-containing DNA.

To test whether the mobility shift was indeed due to binding by the BzpF-GST fusion protein, we added anti-GST antibodies to the binding reaction. We found that the shifted band was super-shifted, indicating that the mobility shift was due to interaction between the oligonucleotide and the fusion protein (Figure 5B, lane 4). We also performed a competition assay by adding unlabeled oligonucleotides to the reaction. Increasing amounts of the mutated oligonucleotide had almost no effect on binding (Figure 5B, right panel, CRE') but increasing amounts of the specific oligonucleotide reduced binding in a dose-dependent manner (Figure 5B, right panel, CRE). These results support the conclusion that the BzpF protein binds CRE in a sequence-specific manner and suggest that BzpF is a candidate CREB protein.

## Discussion

Our experimental results validate our computational approach and indicate that it can discover components of cellular pathways from expression profiles. This work is an extension of work on Bayesian network models using known pathways [33,34]. Our approach improves on these methods by incorporating more complex prior knowledge about the initial core network, including dependencies derived from non-transcriptional data. Our work is similar to other approaches that incorporate various sources of knowledge into the Bayesian framework [35,36], but improved by allowing ranking of thousands of genes to facilitate a more explorative analysis.

The ability of Bayesian networks to discover dependences between more than two genes makes the technique more powerful than co-expression networks. For example, our single-gene-expansion method discovered *egeB*, a gene that encodes a C2-domain-containing cytosolic protein. That gene was highly ranked by our single-gene-expansion method, but not by the co-expression networks. *egeB *is a member of a gene family that is responsible for induction of genes involved in early development [37] . Our analysis found transcriptional dependence between *egeB *and two early developmental genes, *pufA *and *acaA*. Although no direct regulation of *pufA *and *acaA *has been reported, there is evidence for indirect regulation through *yakA *and *carA*, respectively [37]. With the power to detect higher order relationships, our Bayesian networks algorithm detected these interactions, while the co-expression networks approach fell short. Despite this advantage many of the previously characterized genes show higher ranking by co-expression analysis compared with the single-gene expansion method. Most likely the 76 genes identified only by the single-gene expansion method are lowering the rankings of the 133 identified by both methods. We expect the highly ranked genes with multiple dependences to be members of the PKA pathway and exhibit clear developmental phenotypes as we characterize more knockout mutants.

Since the known network is expanded by one gene at a time, our method cannot detect interactions where two or more non-core genes are involved in the regulation. Since bZIP transcription factors are known to heterodimerize and many of them may have overlapping functions [38], the fact that single knockouts of *bzpR *and *bzpG *do not exhibit developmental phenotypes does not exclude them as potential members of the PKA pathway. Expanding the known networks by more than one gene at a time has the potential to identify more interacting partners, but since this extension requires exponentially more computational time it was not implemented for the PKA pathway analysis.

Our algorithm discovers genes that have conditional dependences with members of the PKA pathway, regardless of whether they are members of the PKA-pathway or of parallel pathways. For example, we found the transcriptional regulator *gbfA*, which is considered a member of a parallel pathway [39]. Although PKA activity is not required for *gbfA *induction or activity, maximal mRNA expression of *gbfA *does require PKA activity [39], suggesting conditional dependence between these components.

## Conclusions

Computational methods that infer Bayesian networks can uncover gene expression dependencies in large datasets and thus provide means of proposing gene expression pathways. We introduce a novel strategy for using Bayesian networks, designed for discovering new genes of known genetic networks. This method incorporates prior biological knowledge from many different sources into the structure of the starting network and discovers new components that may have higher-order dependencies with members of the initial network. We applied this method to the PKA pathway in *D. discoideum *and validated the top predictions by performing direct genetic tests. The experimental results identified *dhkL*, a new candidate up-stream regulator of PKA, and *bzpF*, a candidate CREB homologue in *D. discoideum*. Although the initial PKA network does not reflect the underlying transcriptional network, the single-gene-expansion method was successful in identifying new members. Modeling networks that better represent the underlying regulatory network may be even more informative.

The success of this method can be attributed to the power of Bayesian networks and to the nature of our dataset. We propose that successful modeling requires a large dataset representing a wide range of cellular states. The underlying network and probability distribution might be perturbed in some mutant strains and under some experimental conditions, and therefore trying to model the wild type network using a heterogeneous dataset can confound the analysis. On the other hand, perturbation experiments are essential for creating the necessary range of cellular states required for identifying gene interactions. Therefore one must consider the tradeoff between maintaining the wild type network and the information gain from perturbation experiments. We chose the PKA pathway because it plays a central role in all stages of *Dictyostelium *development and our dataset contained many knockout experiments for genes related to the core pathway. Many of these perturbations affect the pathway function during specific stages, and therefore provide the necessary information for detecting gene dependences while maintaining the wild type network during the other stages. Therefore our dataset provides the required resolution for detecting new members of the PKA pathway.

## Authors' contributions

E.H. and C.D. performed the experiments; A.P. performed the data analysis; A.P., D.S. and G.S. wrote the manuscript; all of the authors contributed to the research design, discussed the results, commented on the manuscript and read and approved the final manuscript.

## Supplementary Material

Additional file 1 **Single-gene expansion script**. Matlab script file containing the single-gene expansion algorithm. The matlab BNT toolbox and the expression data are required for the script to run. The BNT toolbox can be downloaded from http://code.google.com/p/bnt/. The complete expression dataset can be downloaded from http://www.ailab.si/dictyexpress/data.htm. Click here for file

Additional file 2 **Supplementary Table S1**. Gene ranked in the top 5% by the single-gene expansion algorithm. Click here for file

Additional file 3 **Supplementary Table S2**. Gene ranked in the top 5% by co-expression analysis. Click here for file
